# Multifunctional nanoplatform based on star-shaped copolymer for liver cancer targeting therapy

**DOI:** 10.1080/10717544.2019.1625467

**Published:** 2019-06-14

**Authors:** Xianling Gong, Yi Zheng, Guangzhi He, Kebing Chen, Xiaowei Zeng, Zhihong Chen

**Affiliations:** a Guangdong Key Laboratory for Research and Development of Natural Drugs, School of Pharmacy, Guangdong Medical University, Zhanjiang, China;; b The Center of Medical Genetics and Molecular Diagnosis, Department of Ultrasound, University of Chinese Academy Sciences-Shenzhen Hospital, Shenzhen, China;; c Department of Orthopedics, The Third Affiliated Hospital of Southern Medical University, Academy of Orthopedics, Guangzhou, China;; d School of Pharmaceutical Sciences (Shenzhen), Sun Yat-Sen University, Guangzhou, China;; e Analysis Centre, Guangdong Medical University, Dongguan, China

**Keywords:** Lactobionic acid, star-shaped copolymer, cancer nanotechnology, nanomedicine, targeting drug delivery

## Abstract

With high morbidity and death rates, liver cancer has become one of the most common cancers in the world. But, most chemotherapeutic anticancer drugs have high toxicity as well as low specificity. To improve the treatment modalities and enhance the therapeutic effect of liver cancer, a brand new liver-targeting nanoparticle (NP), Ent-11α-hydroxy-15-oxo-kaur-16-en-19-oic acid (5 F)-loaded cholic acid (CA)-functionalized star-shaped poly (lactic-*co*-glycolic acid) (PLGA)-polyethylene glycol (PEG)-lactobionic acid (LA) (5 F-loaded CA-PLGA-PEG-LA), was developed. The particle size, zeta potential, size distribution, surface morphology, drug loading content, drug encapsulation efficiency and drug release of 5 F-loaded NPs were characterized. Confocal microscopy and flow cytometry showed that the prepared NPs could be internalized by HepG2 cells. Furthermore, the cellular uptake efficiency of coumarin 6-loaded CA-PLGA-PEG-LA NPs was much better in compare with that of CA-PLGA-PEG and CA-PLGA NPs. Moreover, LA-conjugated NPs (CA-PLGA-PEG-LA NPs) enhanced fluorescence of HepG2 cells via ligand-mediated endocytosis. The antitumor effects of 5 F-loaded NPs were evaluated by the MTT assay *in vitro* and by a xenograft tumor model *in vivo*, demonstrating that targeted 5 F-loaded CA-PLGA-PEG-LA NPs were significantly superior to free 5 F and 5 F-loaded CA-PLGA-PEG NPs. All the results indicated the 5 F-loaded CA-PLGA-PEG-LA NPs can be employed as a novel potentially targeting drug delivery system for liver cancer therapy.

## 1. Introduction

As one of the most common cancers, liver cancer shows high incidence and mortality rates, especially in less developed countries (Tao et al., 2014; Torre et al., 2015; Yang et al., 2018). The treatments of liver cancer mainly include surgical resection, chemotherapy, radiotherapy and immunotherapy. So far, chemotherapy is widely used as therapeutic method in clinical treatment, but most of these chemotherapeutic drugs cannot effectively distinguish between cancer cells and normal cells *in vivo*, which also cause systemic toxicity and side effects. Targeted delivery of anticancer drugs into the specific cells may significantly improve this.

The asialoglycoprotein receptor (ASGPR), a liver-associated surface receptor, is abundantly and specifically present on hepatocytes (Stockert et al., 1974; Ashwell & Harford, 1982; Fiete et al., 1991; Rensen et al., 2001; D'Souza & Devarajan, 2015). The receptor specifically recognizes the end group coupling galactose residues or lactose moieties and combines with it. After the complex is formed, the complex ligand-ASGPR receptor can be quickly internalized. When the complex approaches the lysosomal compartment, the ligand is released. Next, the ligand is degraded in the lysosome and the drug is released. The receptor will be re-oriented toward the plasma membrane (Ashwell & Harford, 1982). So, by special recognition of ASGPR receptor and binding to galactose residues or lactose moieties of NPs, the NPs can exert liver-targeting effect. Previous studies have shown NPs conjugating galactose residues or lactose moieties can effectively and specifically target the sites of hepatoma tumor through the ASGP receptor-mediated recognition (Liang et al., 2006; Craparo et al., 2014; Iacobazzi et al., 2017; Tsend-Ayush et al., 2017).

Poly (lactic-*co*-glycolic acid) (PLGA), an extensively used biomaterial which has been approved by the US FDA, has favorable properties such as good biocompatibility, biodegradability, and mechanical strength (Jain, 2000; Dinarvand et al., 2011). Its degradation is nontoxic in nature and noncumulative *in vivo* (Dinarvand et al., 2011; Pang et al., 2018). PEG is a safe material approved by FDA (Liu et al., 2018; Peng et al., 2018). Surface modification of PLGA-based NPs through PEGylation process can improve its hydrophilicity. The protective hydrophilic layer around the external surface of NPs can repel the adsorption of opsonin proteins by steric repulsion forces, which blocks and delays the binding of opsonins to the surface of the nanoparticle (NP) in the opsonization process. Moreover, the free NPs from the bloodstream can escape phagocytosis of the macrophages of the mononuclear phagocytic system (MPS). So the conjugation of PEG or PEG-containing copolymers on the surface of NPs leads to an enhanced blood circulation half-life of the NPs via several orders of magnitude (Yang et al., 2016; Cheng et al., 2017; Zeng et al., 2018). The preparation techniques of polymeric NPs include nanoprecipitation, solvent evaporation and dialysis (Tao et al., 2013, 2016; Rosenblum et al., 2018; Jamil et al., 2019).

Although the linear PLGA-PEG NPs have been studied as drug delivery vehicle by many researchers, star-shaped copolymers have attracted a great deal of research interest because of excellent properties. Cholic acid (CA), an important natural compound founded in animals, belongs to a steroid containing three hydroxyl groups and one carboxyl group. The poly-hydroxy groups of CA have been used as initiating sites to achieve star-shaped copolymer (Luo et al., 2009; Yang et al., 2015). Compared to the linear polymers with a similar molar mass, CA-based star-shaped copolymers for drug delivery system exhibit certain advantages, such as higher drug loading content (LC) and higher drug encapsulation efficiency (EE) (Cunningham et al., 2018).

*Pteris semipinnata* is a kind of widely used traditional medicinal plant in China. Ent-11α-hydroxy-15-oxo-kaur-16-en-19-oic-acid, which is termed as 5 F simply, is a major bioactive compound that belongs to diterpeniod from *Pteris semipinnata*. Although 5 F suppressed hepatoma tumor growth in mice by intraperitoneal injection obviously (Chen et al., 2012), the small molecular drugs were randomly distributed *in vivo* and most of them were excreted *in vitro*, which led to low bioavailability. In order to improve bioavailability and enhance the therapeutic efficacy of 5 F, it is necessary to prepare 5 F-loded NPs for targeting liver.

Lactobionic acid (LA), which is composed of one molecule of galactose attached to one molecule of gluconic acid through an ether-like linkage, belongs to multiple hydrophilic group oligosaccharide (Belkacemi & Hamoudi, 2010). 5 F-loaded CA-PLGA-PEG NPs, the star-shaped amphiphilic block copolymers, were surface-modified with LA, a ligand whose galactosyl residue specifically couples with the asialoglycoprotein receptor (ASGPR), allowing the targeted delivery of drugs to liver cancer cells.

In the present study, using CA as initiator, we developed a novel star-shaped amphiphilic 5 F-loaded CA-PLGA-PEG-LA NPs. LC, EE, morphology, *in vitro* drug release behavior, *in vitro* cellular uptake, and cytotoxicity of the NPs were tested.

## 2. Materials and methods

### 2.1. Materials

4-Dimethylaminopyridine (DMAP), *N*-hydroxysuccinimide (NHS), 1-ethyl-3-(3-dimethylaminopropyl)carbodiimide (EDC), d-α-tocopheryl polyethylene glycol 1000 succinate (TPGS), LA and coumarin 6 were bought from Sigma Aldrich (St. Louis, MO, USA). 5 F, whose purity is more than 98% (HPLC Grade), was from Guangdong Key Laboratory for Research and Development of Natural Drugs (Zhanjiang), Guangdong Medical University. Star-shaped copolymers CA-PLGA (*M*_w_ ≈ 15,000) and CA-PLGA-PEG-NH_2_ (*M*_w_ ≈ 21,000) was kindly provided by School of Pharmaceutical Sciences (Shenzhen), Sun Yat-sen University. Methanol for HPLC grade were purchased from Merck & Co., Inc. (Germany).

### 2.2. Synthesis of 5 F-loaded CA-PLGA-PEG-NH_2_ and CA-PLGA-PEG-LA NPs

5F-loaded CA-PLGA-PEG-NH_2_ NPs were achieved according to the previous method (Gao et al., 2015; Zeng et al., 2015). 5F and copolymer CA-PLGA-PEG-NH_2_ were dissolved in dichloromethane. The organic solution was slowly poured into aqueous phase containing TPGS under stirring. The mixture was sonicated (2 min, 25 W) to form an oil/water emulsion. The excess dichloromethane was removed by stirring for 4 h. The obtained dispersions of NPs were centrifuged (15 min, 20,000 rpm), and then washed with distilled water for 3 times to eliminate the emulsifier TPGS and then lyophilized for 2 days. 5 F-loaded CA-PLGA-PEG-NH_2_ NPs were obtained. 5 F-loaded CA-PLGA and fluorescent coumarin 6 (C6)-loaded NPs were prepared by the similar procedure.

5 F-loaded CA-PLGA-PEG-LA NPs were fabricated by coupling reaction of 5 F-loaded CA-PLGA-PEG NPs and LA in the presence of EDC and a catalytic amount of NHS.

### 2.3. Size, zeta potential and morphology of the NPs

The size and zeta potential of NPs were examined using Malvern Mastersizer 2000 Particle Analyzer (Zetasizer Nano ZS90, Malvern Instruments Ltd., UK). The freshly prepared NPs were diluted with distilled water. After equilibration for 10 min, the detections were performed at 25 °C. The data were obtained with the average of three measurements.

The morphology of NPs was characterized by transmission electron microscopy (TEM, Tecnai G2 20, FEI Company, Hillsboro, Oregon, USA). The NPs power was suspended by deionized water at first. Then the solution of NPs was dropped onto a copper grid coated with a carbon membrane and dried at room temperature before observing the TEM images.

### 2.4. Drug loading and encapsulation efficiency

High-pressure liquid chromatography (HPLC) (LC 1200; Agilent Technologies, CA, USA) was used to measure the amount of 5 F as drug loading or encapsulation efficiency. In brief, 5 F-loaded NPs were dissolved in 1 ml dichloromethane by vigorous vortexing. The solution was added to 5 ml of mobile phase comprising methanol and distilled water at a ratio of 55:45 (V/V). A nitrogen stream was supplied constantly to evaporate dichloromethane for about 15 min. Filter was performed to get rid of precipitation. The resulting clear solution was subject to HPLC analysis with a reverse-phase C18 chromatographic column (4.6 × 150 mm, 5 μm; Agilent Technologies, CA, USA) at 25 °C. The flow rate of mobile phase was 1 ml·min^−1^. The measured wavelength was 242 nm. The measurement was performed in triplicate.

### 2.5. *In vitro* drug release study

*In vitro* drug release study of 5 F from drug-loaded NPs was monitored with a dialysis method reported previously (Zeng et al., 2015). 5 mg lyophilized NPs were dispersed in 5 ml phosphate buffer solution (PBS, PH = 7.4) containing 1% (w/v) Tween 80. The suspension was added into a dialysis bag (MWCO: 3500 Da, Spectra/Por 6; Spectrum Laboratories, USA). The sealed dialysis bag was immersed in 15 ml of PBS release medium and incubated in a thermostatic shaker at 120 rpm at 37 °C. At indicated time, quantitative receiver medium was withdrawn and replaced with fresh medium. The 5 F release amount in each sample was detected via HPLC analysis mentioned above.

### 2.6. Cell line and cell culture

Human hepatocellular carcinoma cell line HepG2 and human colon carcinoma cell line SW480 cell were bought from the Chinese Academy of Science Committee Type Culture Collection Cell Bank (Shanghai, China). HepG2 cells were incubated in DMEM medium (Gibco) and SW480 cells in RPMI 1640 medium (Gibco). The medium was supplemented with 10% fetal bovine serum (Gibco) and 1% penicillin-streptomycin (Invitrogen). Cells were grown at 37 °C in a humidified incubator of 5% CO_2_-containing atmosphere.

### 2.7. Cellular uptake of C6-loaded NPs

Coumarin 6 (C6), a model fluorescent maker, was encapsulated in the CA-PLGA NPs, CA-PLGA-PEG NPs, and CA-PLGA-PEG-LA NPs to investigate the uptake by HepG2 cells.

The cells were seeded in 35 mm glass-bottom micro-well culture dish. After incubated overnight, the cells were incubated with 100 μg·ml^−1^ C6-loaded CA-PLGA NPs, C6-loaded CA-PLGA-PEG NPs and C6-Loaded CA-PLGA-PEG-LA NPs for 2 h, respectively. The medium was discarded. Cells were washed with PBS for three times and then fixed with 4% paraformaldehyde at room temperature for 25 min. Then cell nuclei were stained by DAPI for 10 min. The imagines were captured by confocal microscope (NIKON C2^+^).

For flow cytometric analysis, the cells were seeded in six-well plates, cultured overnight and next treated with 100 μg/ml C6-loaded CA-PLGA NPs, C6-loaded CA-PLGA-PEG NPs and C6-loaded CA-PLGA-PEG-LA NPs for 2 h, respectively. Then they were rinsed three times with cold PBS, detached with trypsin, centrifuged at 800 rpm for 5 min and resuspended in PBS. Finally, the intracellular fluorescence of C6 was measured using flow cytometry (BD FACSCalibur, San Jose, CA, USA).

### 2.8. *In vitro* toxicity of 5 F-loaded NPs

Human hepatocellular carcinoma cells HepG2 were seeded at density of 5 × 10^3^ cells per well in 96 wells plates. On the next day, cells were treated with different concentration of drug-free CA-PLGA-PEG-LA NPs, 5 F, 5 F-loaded CA-PLGA NPs, 5 F-loaded CA-PLGA-PEG NPs, 5 F-loaded CA-PLGA-PEG-LA NPs for various time. MTT (3-[4,5-dimethylthiazolyl-2]- 2,5-diphenyl tetrazolium bromide) (MP, France) was added into each well. Cells were cultured for 4 h. The growth medium was removed. The formazan crystals were dissolved in DMSO. The absorbance value was measured by a microplate reader (Thermo, USA) at 490 nm.

### 2.9. *In vivo* antitumor efficacy of 5 F-loaded NPs

Female nude mice (BALB/c-nu, 5–6 weeks old) were bought from Guangdong Medical Laboratory Animal Center (China). All the protocols for the proposed *in vivo* experiments were approved by the Administrative Committee on Animal Research in Sun Yat-sen University. Guidelines of the institutional animal ethics committee were followed for *in vivo* experiments. Each mouse was subcutaneously injected with 100 μL of medium containing about 2 × 10^6^ HepG2 cells into the right axilla. Tumors were measured by a vernier caliper and its volume (V) was calculated as *V* = *d*^2^×*D*/2, where *d* and *D* are shortest and the longest diameter of the tumor in mm, respectively. Treatment was performed when the volume reached approximately 80 mm^3^ (referred to as the 0th day). The tumor-bearing mice were divided into five groups randomly (*n* = 5) and subjected to tail intravenous injection with saline, drug-free CA-PLGA-PEG-LA NPs, 5 F, 5 F-loaded CA-PLGA-PEG NPs, and 5 F-loaded CA-PLGA-PEG-LA NPs with a 5 F dosage of 10 mg kg^−1^ (equivalent 5 F dosage for 5 F-loaded NPs). The injection was conducted every 4 days. The tumor volume and body weight of the treated-mice were recorded every other day until the end of the treatment. In order to further investigate the antitumor effect of 5 F-loaded NPs, the histological analysis was performed. Tumors were excised and fixed in 10% neutral buffered paraformaldehyde overnight. Thereafter, the tumors were embedded in paraffin and stained with hematoxylin and eosin (H&E) and observed by optical microscope.

### 2.10. Statistical analysis

The statistical analysis of the data was carried out by SPSS 22.0 statistical software. And the results were reported as mean ± standard deviation (SD). Comparisons were performed using one Way-ANOVA followed by least-significant difference (*LSD*) correction for multiple tests. A *p* < .05 was considered as statistically significant difference. Parallel experiments were repeated three times.

## 3. Results and discussion

### 3.1. Characterization of NPs

**Figure 1.**
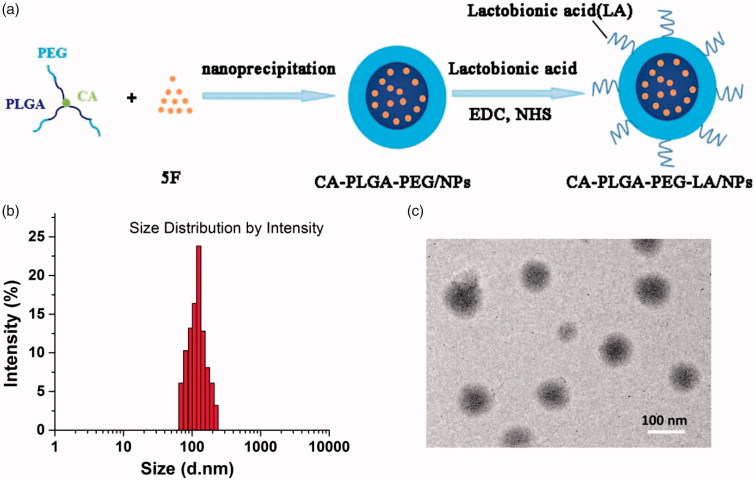
The synthesis and characterizations of 5 F-loaded CA-PLGA-PEG-LA NPs. (a) Schematic illustration of synthesis procedure of the NPs. (b) DLS size distribution of the NPs. (c) TEM imagine of the NPs.

We prepared the coumarin 6-loaded or 5 F-loaded NPs of CA-PLGA, CA-PLGA-PEG and CA-PLGA-PEG-LA. The synthesis process of 5 F-loaded NPs was shown as Figure 1(a). The copolymer and drug could be completely dissolved in dichloromethane to form a homogenous and clear solution. Then, the organic solution was slowly added into aqueous solution under stirring. Meanwhile, a rapid precipitation of the hydrophobic segment PLGA of the block copolymer occurs, resulting in spontaneous formation of drug entrapped CA-PLGA-*b*-PEG-NH_2_ NPs. Then, a stable drug-loaded NPs aqueous dispersion was obtained after stirring to remove organic solvent dichloromethane. Finally, the NPs have a core-shell structure with hydrophobic PLGA as the core entrapping water-insoluble drug, and PEG segment as the hydrophilic stabilization shell (Zeng et al., 2013; Tao et al., 2015). The particle size and surface properties are crucial factors which affect drug release, *in vitro* cellular uptake, *in vivo* biodistribution and pharmacokinetics of NPs (He et al., 2010; Kulkarni & Feng, 2013; Kore et al., 2014). Dynamic light scattering (DLS) method was utilized to examine the particle sizes and size distributions of the 5 F-loaded NPs, and the data are presented in Table S1. The average diameter was 148.1 ± 12.9 nm, 120.4 ± 8.5 nm, and 123.8 ± 9.1 nm for 5 F-loaded CA-PLGA, CA-PLGA-PEG, and CA-PLGA-PEG-LA NPs, respectively. Figure 1(b) exhibited the size distribution of the 5 F-loaded CA-PLGA-PEG-LA NPs. The hydrodynamic sizes of NPs synthesized in our study were 100–170 nm, which is within the optimal size range for cellular uptake (Padhi et al., 2018). As a result, they can easily accumulate in the sites of tumor vasculature, resulting in passive tumor targeting effect due to the enhanced permeability and retention effect (Win & Feng, 2005). The polydispersity index of all NPs was less than 0.2 which displayed a narrow size distribution and was beneficial to drug delivery systems.

Zeta potential can measure the intensity of mutual exclusion or attraction between particles and is significant for the physical stability of the NPs. We detected the zeta potential of the 5 F-loaded NPs and the data were listed in Table S1. The values were −29.2 ± 3.7, −14.6 ± 2.5 and −12.0 ± 2.8 mV, respectively. All NPs were found to be negatively charged, which ascribed to the ionized carboxyl groups of polylactic acid and polyglycolic acid segments (Ma et al., 2010). The modified hydrophilic PEG segments decreased the absolute value of Zeta potential because of their surface charge-shielding effect (Chen et al., 2017; Nie et al., 2017).

To further assess the morphology of 5 F-loaded CA-PLGA-PEG-LA NPs, The NPs was examined by transmission electron microscope (TEM). As presented in Figure 1(c), the NP of 5 F-loaded CA-PLGA-PEG-LA were near-spherical in shape. Its mean size was around 90 nm, which was clearly smaller than that measured by DLS method. Such difference might mainly result from the shrink and collapse effect of NPs in the dry state.

The drug LC of 5 F-loaded CA-PLGA-PEG-LA NPs was higher than that of 5 F-loaded CA-PLGA NPs. The drug EE of 5 F-loaded CA-PLGA, CA-PLGA-PEG and CA-PLGA-PEG-LA NPs was 81.5%, 98.7% and 95.7%, respectively (Table S1). 5 F-loaded CA-PLGA-PEG-LA and CA-PLGA-PEG NPs had similar drug LC and EE. These results suggested that the hydrophobic drug 5 F and them had the stronger binding respectively and so could show more excellent characters for clinical applications.

### 3.2. Stability of 5 F-loaded NPs

**Figure 2.**
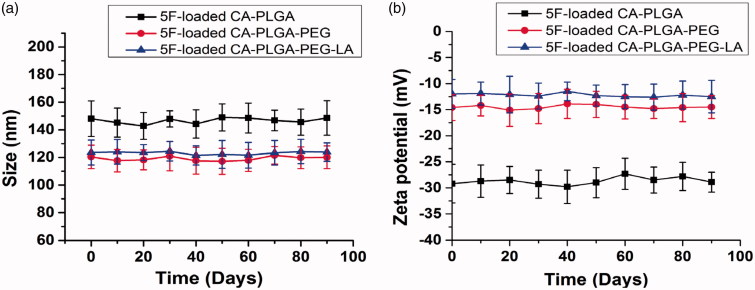
Stability of 5 F-loaded NPs *in vitro*. (a) Particle size and (b) zeta potential of 5 F-loaded CA-PLGA, CA-PLGA-PEG and CA-PLGA-PEG-LA NPs during 90 days of storage, respectively.

When NPs are stored, NPs tend to aggregate because of decrease of the absolute value of zeta potential. For this reason, it is important to keep stability of NPs for therapy. To observe the stability of 5 F-loaded NPs which were stored at 4 °C, their average size and zeta potential were detected every 10 days after preparation. As exhibited in Figure 2, the size or zeta potential of 5 F-loaded NPs showed no obvious change at 4 °C during 90 days of storage. The data demonstrated that the NPs were fairly stable.

### 3.3. *In vitro* drug release profile

**Figure 3.**
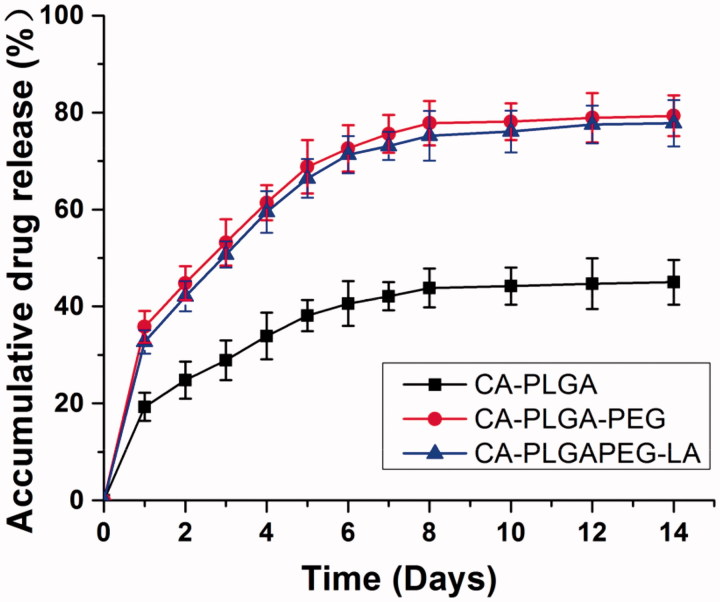
The *in vitro* drug release behavior of 5 F-loaded CA-PLGA, CA-PLGA-PEG and CA-PLGA-PEG NPs. Error bars represent standard deviation (SD) for *n* = 3.

Figure 3 displayed the *in vitro* drug release profiles of 5 F-loaded NPs in PBS (PH = 7.4) with 0.1% (w/v) Tween 80 for 14 days. During the first 2 days, the initial burst release of 5 F-loaded CA-PLGA, CA-PLGA-PEG and CA-PLGA-PEG-LA NPs was found to be 24.8%, 44.8% and 42.1%, respectively. But over the following days, all NPs released 5 F in a sustained and continuous means. After14 days, their accumulative drug releases were 45.0%, 79.3% and 77.8%, respectively. These results suggested that the drug release showed a typically biphasic pattern with a small initial burst. The burst release could result from that 5 F was only encapsulated beneath the periphery of NPs. Afterwards, 5 F was steadily released mainly owing to the diffusion of the drugs that were well loaded in the hydrophobic inner shell of NPs or from rigid core. The drug release from polymeric NPs was a diffusion process, so the NPs with higher density and drug LC more easily release drug. With the decreasing density of drugs inside the inner shell, the drug release decelerated. Similar results have been reported by Su et al. (2017).

### 3.4. Cellular uptake of coumarin 6-loaded NPs

**Figure 4.**
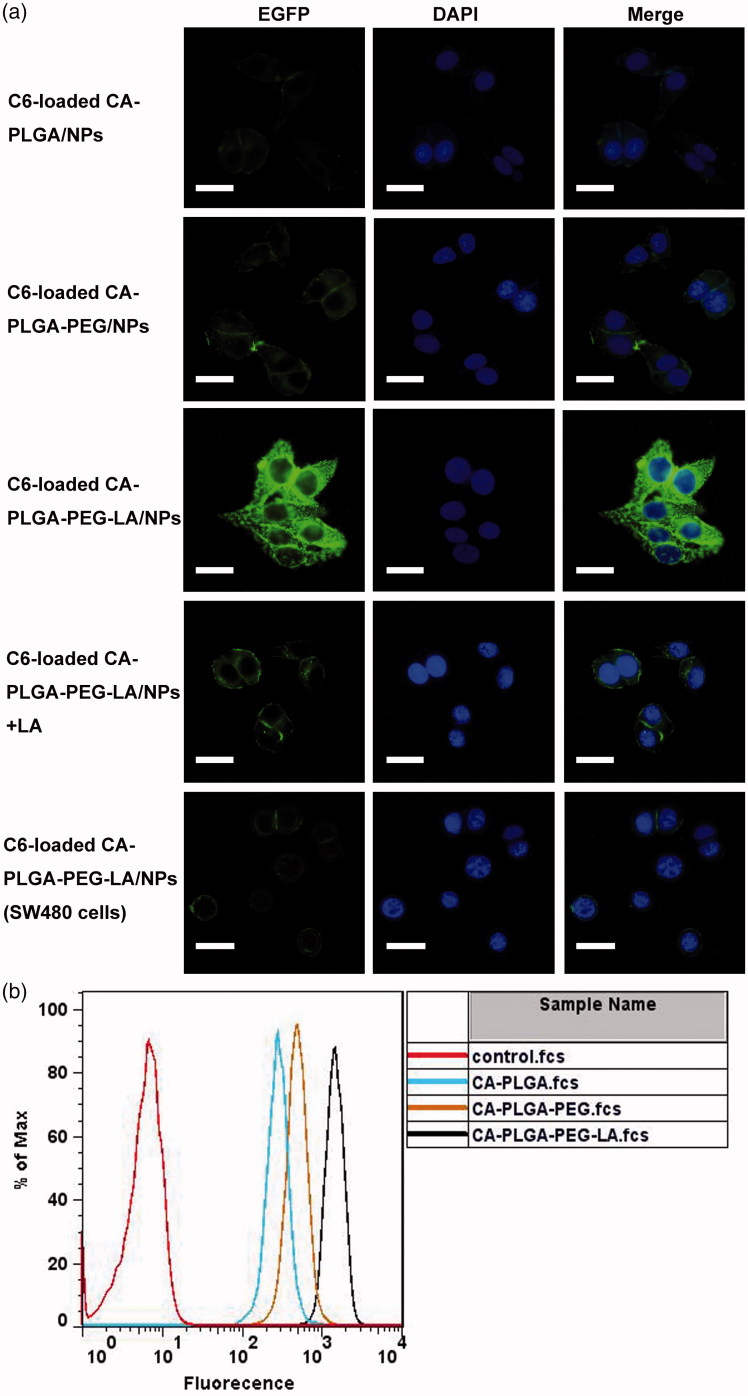
Cellular uptake assay of coumarin-6 loaded NPs. (a) After HepG2 cells were incubated with coumarin-6 loaded NPs for 2 h and then stained by DAPI, the fluorescent images were observed using confocal microscope. Scale bar: 50 μm. (b) Flow cytometry histogram profiles of C6-loaded NPs on HepG2 cells.

Internalization and persistent retention of drug-loaded NPs by cancer cells determine therapeutic effects on cancer (Zhu et al., 2016). Coumarin 6, which is a fluorescent probe, substituted for 5 F to examine the cellular uptake of NPs in HepG2 cells (Zhao & Feng, 2014). The cells were treated with various C6-loaded NPs respectively for 2 h. As displayed in Figure 4(a), the cytoplasm of HepG2 cells showed green fluorescence, which dispersed around the blue nuclei stained by DAPI. However, C6-loaded CA-PLGA-PEG-LA NPs treatment resulted in significantly enhanced fluorescence intensity in the cytoplasm as compared with C6-loaded CA-PLGA and CA-PLGA-PEG NPs-treated HepG2 cells. These results demonstrated C6-loaded CA-PLGA-PEG-LA NPs could be more effectively internalized into HepG2 cells. LA on the surface of CA-PLGA-PEG-LA NPs is necessary for the liver-targeting drug delivery. To examine the role of LA in the internalization process of CA-PLGA-PEG-LA NPs, LA was used as a competitive reagent and C6-loaded CA-PLGA-PEG-LA NPs were added to the same wells simultaneously, fluorescence intensities of HepG2 cells were obviously decreased. Furthermore, little fluorescence was observed in human colon carcinoma SW480 cells were treated with the LA-conjugated NPs (Figure 4(a)). The data suggested the LA-conjugated NPs could specifically interact with HepG2 cells via ligand-receptor (the asialoglycoprotein receptor, ASGPR) recognition.

The cellular uptake efficiency of C6-loaded NPs was further detected by flow cytometry. Using the cells without the treatment of C6 as a control, they only presented autofluorescence from flow cytometry histogram. But the C6-CA-PLGA-PEG-LA NPs displayed higher fluorescence intensity (Figure 4(b)). So, more C6-CA-PLGA-PEG-LA NPs were internalized into cells and the drug concentration increased. The data also validated that improvement of the cellular uptake efficiency of NPs was ascribed to LA group that targeted to HepG2 cells by ASGPR. The results were consistent with the previous research (Dong et al., 2017; Shen et al., 2018).

### 3.5. *In vitro* toxicity of 5 F-loaded NPs

**Figure 5.**
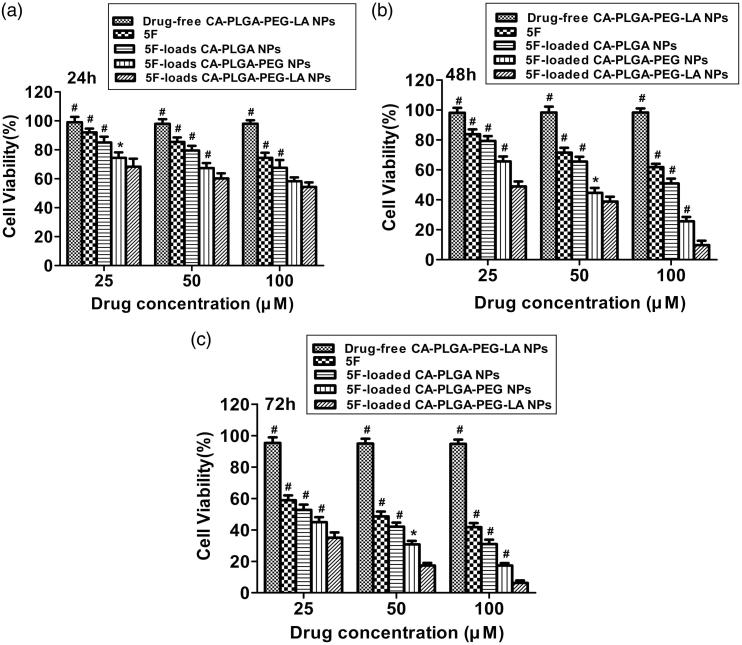
Cell viability of HepG2 treated with drug-free CA-PLGA-PEG-LA NPs, 5 F and 5 F-loaded NPs, respectively. (a) 24 h, (b) 48 h and (c) 72 h. The dosage of 5 F is same in the same group. The amount of drug-free CA-PLGA-PEG-LA NPs was the same as that of the NPs. *p < .05 and #*p* < .01 indicate significant difference compared with 5 F-loaded CA-PLGA-PEG-LA NPs.

In order to assess *in vitro* cytotoxicity of 5 F-loaded NPs, human hepatocellular carcinoma cells, HepG2, were incubated with 5 F-loaded NPs and 5 F at equivalent 5 F concentrations of 25, 50, and 100 µM for indicated time, respectively. Then cell viability was examined using 3-[4, 5-dimethylthiazolyl-2] -2, 5-diphenyl tetrazolium bromide (MTT) assay. Drug-free NPs of different concentrations hardly showed cytotoxicity at various time, which suggested NPs had good biocompatibility. 5 F and 5 F-loaded NPs reduced cell viability of HepG2 in dose-dependent and time-dependent manner. But 5 F-loaded NPs exhibited higher cytotoxicity than 5 F did against HepG2. In addition, inhibition effect of 5 F-loaded CA-PLGA-PEG-LA NPs on HepG2 cells were higher than that of 5 F-loaded CA-PLGA NPs and 5 F-loaded CA-PLGA-PEG NPs (Figure 5). Therefore, 5 F-loaded CA-PLGA-PEG-LA NPs showed the best *in vitro* anti-tumor efficacy on HepG2 for the three NPs. These results indicated 5 F-loaded CA-PLGA-PEG-LA NPs were safe and highly biocompatible and it could be a promising liver-targeting drug.

### *3.6. In vivo* antitumor efficacy

**Figure 6.**
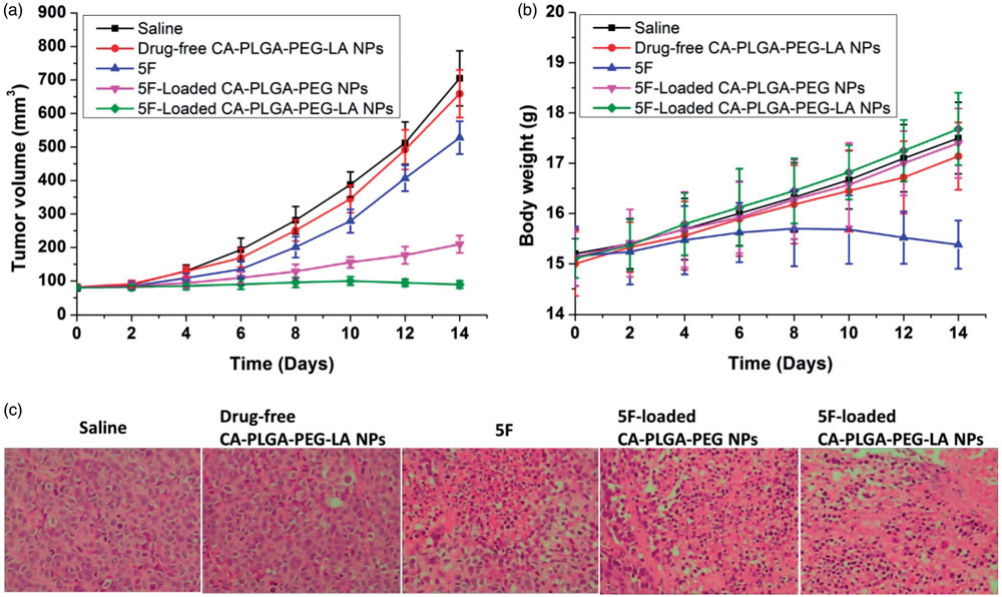
(a) Mean tumor volumes and (b) body weights of the mice in different groups after treatment at different time intervals (*n* = 5). Tail intravenous injection into the HepG2 tumor-bearing mice every 4 days; (c) Representative tissue sections of mice in different groups stained with hematoxylin and eosin (H&E) after 14 days of treatment (Magnification 100×).

Due to the satisfactory cytotoxicity against Hepatocellular carcinoma HepG2 cells *in vitro*, 5 F-loaded CA-PLGA-PEG-LA NPs could be a potential nanocarriers for liver cancer therapy. In this study, the antitumor effects of 5 F-loaded NPs on HepG2 cells-bearing nude mice were also evaluated. Figure 6(a) shows the 2 weeks tumor growth of mice injected with saline (control), drug-free CA-PLGA-PEG-LA NPs, 5 F, 5 F-loaded CA-PLGA-PEG NPs and 5 F-loaded CA-PLGA-PEG-LA NPs. The mice were subjected to tail intravenous injection with samples for every four days. The tumor volume and body weight of mice were recorded until the end of the course (14th day). Finally, the nanoplatform of 5 F-loaded CA-PLGA-PEG-LA NPs possessed of the optimal therapeutic effect and could inhibit the tumor significantly. In the development of novel nanocarriers, it is important to reduce the side effects of drug-loaded nanocarriers. The general side effects on animals can be evaluated by measuring body weight changes. Figure 6(b) shows changes in the body weights of all nude mice. The group of free 5 F treated lost weight remarkably, whereas the mice injected with 5 F-loaded CA-PLGA-PEG NPs and 5 F-Loaded CA-PLGA-PEG-LA NPs gained weight. As can be seen from the experiment, the mice treated by 5 F-loaded CA-PLGA-PEG-LA NPs remained healthy and vigorous, but those treated by free 5 F presented weakened vitality, even one of the mice died. In order to further evaluate the anticancer activity of drug-loaded nanocarriers, tumors were excised from mice on the 14th day and sectioned for H&E analyses and the results were presented in Figure 6(c). The tumor tissues in 5 F-loaded CA-PLGA-PEG and 5 F-loaded CA-PLGA-PEG-LA NPs group showed significant tumor necrosis as compared to other groups. Moreover, the 5 F-loaded CA-PLGA-PEG-LA NPs group had the best therapeutic effect. Therefore, all these results suggested that the 5 F-loaded CA-PLGA-PEG-LA NPs could be used as a potential targeted drug delivery system for liver cancer therapy.

## 4. Conclusions

5F-loaded CA-PLGA-PEG-LA NPs for liver cancer therapy were developed by a slightly modified technique of solvent evaporation. Their average diameter, close to that of 5 F-loaded CA-PLGA-PEG NPs, was around 120 nm using DLS method. Furthermore, size of these two NPs was smaller compared with that of 5 F-loaded CA-PLGA NPs. Moreover, they displayed higher drug LC, EE and faster drug release than those of CA-PLGA NPs. All NPs had a narrow size distribution (<0.2) and exhibited good stability. The proper size and LA ligands could boost drug passive accumulation and active targeting, so the cellular uptake efficiency of CA-PLGA-PEG-LA NPs was obviously superior to those of the other two NPs. Compared with 5 F-loaded CA-PLGA-PEG, 5-loaded CA-PLGA NPs and 5 F, the 5-loaded CA-PLGA-PEG-LA NPs showed the most obvious anti-cancer effect on *in vitro* HepG2 cells and *in vivo* experiment, while drug-free CA-PLGA-PEG-LA NPs were nontoxic. In conclusion, LA-based conjugate modification of 5 F-loaded star-shaped CA-PLGA-PEG-LA NPs have a potential for liver cancer targeting therapy.

## Supplementary Material

Supplementary_Information.docx
