# Supplementary material for: Multifunctional nanoplatform based on star-shaped copolymer for liver cancer targeting therapy
Source: Drug Deliv. 2019 Jun 14;26(1):595–603. doi: 10.1080/10717544.2019.1625467 (PMC6586121; doi:10.1080/10717544.2019.1625467)
Supplement: Supplementary_Information.docx [file IDRD_A_1625467_SM2481.docx]

***Supplementary Information***

**Multifunctional Nanoplatform Based on Star-shaped Copolymer for Liver Cancer Targeting Therapy**

Xianling Gong^a,#^, Yi Zheng^c,#^, Guangzhi He^c^, Kebing Chen^d,^*, Xiaowei Zeng^e,^*, Zhihong Chen^a,b,^*

*^a^ Guangdong key Laboratory for Research and Development of Natural Drugs, School of Pharmacy, Guangdong Medical University, Zhanjiang 524023, China;*

*^b^ Analysis Centre, Guangdong Medical University, Dongguan 523808, China;*

*^c^ The Center of Medical Genetics and Molecular Diagnosis, Department of Ultrasound, University of Chinese Academy Sciences-Shenzhen Hospital, Shenzhen 518107, China;*

*^d^ Department of Orthopedics, The Third Affiliated Hospital of Southern Medical University, Academy of Orthopedics, Guangdong Province, Guangzhou 510630, China;*

*^e^ School of Pharmaceutical Sciences (Shenzhen), Sun Yat-Sen University, Guangzhou 510275, China.*

^#^ These authors contributed equally to this work.

^*^Corresponding author.

E-mail: [chenkebing.sysu@gmail.com](mailto:chenkebing.sysu@gmail.com) (K. Chen)

E-mail: [zengxw23@mail.sysu.edu.cn](mailto:zengxw23@mail.sysu.edu.cn) (X. Zeng)

E-mail: zhchen201@163.com (Z. Chen)

**Table S1** Characterization of 5F-loaded NPs.

| **Polymer** | **Size(nm)** | **PDI** | **ZP(mV)** | **LC(%)** | **EE(%)** |
| --- | --- | --- | --- | --- | --- |
| CA-PLGA | 148.1 ± 12.9 | 0.183 | -29.2 ± 3.7 | 8.2 | 81.5 |
| CA-PLGA-PEG | 120.4 ± 8.5 | 0.155 | -14.6 ± 2.5 | 9.5 | 98.2 |
| CA-PLGA-PEG-LA | 123.8 ± 9.1 | 0.146 | -12.0 ± 2.8 | 9.1 | 95.7 |

PDI = Polydispersity index, ZP = Zeta potential, LC = Loading content, EE = Encapsulation efficiency, *n* = 3.
